# Epi-off-lenticule-on corneal collagen cross-linking in thin keratoconic corneas

**DOI:** 10.1007/s10792-020-01526-x

**Published:** 2020-08-13

**Authors:** Carlo Cagini, F. Riccitelli, M. Messina, F. Piccinelli, G. Torroni, D. Said, A. Al Maazmi, H. S. Dua

**Affiliations:** 1 Department of Biomedical and Surgical Sciences, Ophthalmology Section, University of Perugia, Perugia, Italy; 2 Larry A Donoso Laboratory for Eye Research, Division of Clinical Neuroscience, Academic Section of Ophthalmology, University of Nottingham, Nottingham, UK

**Keywords:** Keratoconus, Thin cornea, Collagen cross-linking, Treatment

## Abstract

**Purpose:**

To evaluate the safety and efficacy of corneal collagen cross-linking (CXL) performed on overlaying a corneal lenticule to thin recipient corneas of progressive keratoconus (KC) patients.

**Methods:**

In this study were enrolled eyes of patients affected by progressive KC with a minimum corneal thickness less than 400 µm, after overlaying a lenticule of human corneal stroma prepared with the femtosecond laser. The lenticules used were 100 µm thick and of 8.5 mm diameter in all the cases. Both the host cornea and the lenticules were subjected to epithelial debridement. CXL was carried out according to the standard protocol. Visual acuity, refraction, slit-lamp examination, endothelial cell density, pachymetry and keratometry, anterior segment tomography (AS-OCT) and confocal microscopy were evaluated preoperatively and at 1, 3, 6 and 12 months postoperatively.

**Results:**

CXL was performed in 10 eyes of 8 patients (main age 23), corneal thickness range 379–414 µm, mean 387.6 µm. One patient was lost at follow-up. In all other cases, visual acuity and the endothelial cell density remained stable over a 12-month follow-up. Preoperative mean K1 and mean K2 were 46.91 ± 1.9 and 50.75 ± 2.93, respectively, and at 12 months mean K1 was 47.36 ± 2.66 and mean K2 50.53 ± 3.35. The AS-OCT clearly showed a demarcation line in all patients at 1, 3 (mean depth 283 µm and 267 µm, respectively) and in some cases at 6 months. Reduced keratocyte density and stromal oedema were observed immediately up to 1 month after treatment, while a slight subepithelial haze was present at 1-month and completely disappeared by 6 months.

**Conclusion:**

This new technique seems to offer a therapeutic opportunity for young patients suffering from progressive KC with very thin corneas, in which the standard treatment is not indicate, and delay or avoid the need for a corneal transplant.

## Introduction

Keratoconus (KC) is a progressive, non-inflammatory, bilateral asymmetric disease of the cornea characterized by steepening and distortion of its shape, resulting in decreased vision secondary to irregular astigmatism, scaring and progressive myopia. The pathophysiologic events leading to the weakening, thinning and ectasia of the cornea are not completely understood [1].

Corneal collagen cross-linking (CXL) is an intervention that has been shown to be effective in arresting or slowing the progression of ectasia. A variety of protocols have been suggested, which include conventional CXL, accelerated CXL, contact lens-assisted CXL, transepithelial protocols with and without iontophoresis [2–5].

CXL is the gold standard technique for corneal stabilization in KC but when the thickness is less than 400 µm, the procedure is contraindicated because the risk of ultraviolet-A (UVA) damage to the endothelium and other intraocular structures is high [6]. Some CXL techniques have been described to overcome this limit as transepithelial CXL, custom epithelial debridement or increasing cornea thickness by the use of hypo-osmolar solutions, but none of them has reached a high diffusion due to the variability of the results obtained and to the dubious safety of the treatment. Some authors to treat thin corneas described a technique of stromal expansion using a donor lenticule obtained after small incision lenticule extraction (SMILE) for myopic correction [7]. In this study, we present a modified approach to CXL in progressive KC with less than 400 µm corneal thickness, wherein a stromal lenticule of standard thickness, obtained by femtosecond laser dissection from a donor cornea, is overlaid on the thin cornea after removal of the epithelium. This increases the thickness, bringing it to the safe range for successful conventional CXL to be carried out. To our knowledge, there are no similar studies in the literature.

## Subjects and methods

The study was approved as a service evaluation audit NO 17-200H in accordance with Nottingham University Hospitals Trusts and was conducted in accordance with the tenets of the Declaration of Helsinki, and all patients signed a written informed consent form before the surgery. Patients with advanced and in progression KC and corneal thickness less than 400 µm were included in the study Corneal thickness was evaluated with the corneal topography (Sirius topographer CSO, Florence, Italy) and the same instrument was used to document the progression of the ectasia 6 months post the treatment. All the patients were contact lens wearers.

Patients with a history of previous corneal infection including herpetic keratitis, dry eye, concomitant ocular or systemic autoimmune disease and the presence of central or paracentral opacities were excluded. All patients were required to discontinue contact lenses for a minimum of 4 weeks before the baseline evaluation. Visual acuity, slit-lamp examination, endothelial cell density (Tomey USA’s EM 3000 Nishi-Ku, Nagoya, Aichi-ke, Japan), corneal topography (Sirius topographer CSO, Florence, Italy) anterior segment optical coherence tomography (AS-OCT) (HRA II, Heidelberg Engineering GmbH, Dossenheim, Germany) and in vivo confocal microscopy (IVCM) (Heidelberg Retina Tomograph II— Rostock Corneal Module, Heidelberg Engineering GmbH, Dossenheim, Germany) examinations were performed preoperatively and 1, 3, 6 and 12 months postoperatively. The evaluation of visual acuity has always been performed with a contact lens.

The lenticule for stromal expansion was prepared the same day of the CXL using a cornea sent from the eye bank which was not suitable for penetrating or endothelial keratoplasty due to low endothelial cell count. The lenticule was prepared with a Visumax femtosecond laser (Carl Zeiss Meditec, Oberkochen, Germany) after positioning the cornea on an artificial anterior chamber. The corneal epithelium was removed by mechanical brushing when a proper pressure was obtained in the artificial anterior chamber with the right height of the infusion bottle. The stromal lenticule was finally prepared excising in all cases the anterior stroma, including the Bowmans membrane, with the femtosecond laser. The lenticule thickness was standardized to 100 µm and 8.5 mm diameter in all cases. The recipient central corneal epithelium was marked as a circle of 8.5 mm and removed by mechanical brushing, and the lenticule was placed on the donor cornea. One drop of riboflavin (Sooft, Fermo, Italy) was applied every 5 min for 30 min and one drop every 5 min under UVA radiation for the next 30 min using the VEGA CBM-X- Linker (CSO, Florence, Italy) following the Dresden protocol (370 nm UVA exposure at 3 mW cm^2^ [8]_._

At the end of the procedure, the lenticule was removed, and in all cases, we applied a contact lens and medication with chloramphenicol/dexamethasone (Betabioptal, Thèa Farma, S.p.A., Milan, Italy).

And moxifloxacin (Vigamox, Alcon Italy, S.p.A., Milan, Italy) eye drops was administered. Postoperative therapy was ofloxocin (Exocin, Allergan, Inc., Dublin, Ireland) eyedrops 1 drop four times daily for seven days, netilmicin/dexamethasone (Netildex, SIFI, S.p.A., Catania, Italy) eyedrops 1 drop four times daily for 15 days, hyaluronic acid (BluYal, Sooft, S.p.A. Montegiorgio, Italy) eyedrops 1 drop four times daily for 30 days, while contact lens removal was done on the third/fifth day at complete reepithelization.

### Statistical analysis

Statistical analysis was completed using IBM SPSS Statistics 24 (SPSS Inc., Somers, NY, USA) and was done by the one-way ANOVA with Dunnett’s ad hoc correction.

## Results

Ten eyes of 8 patients were included in the study, main age 23 years + 3.2 (range 15–28), 8 male and 2 females, with corneal thickness range from 367 to 414 µm (mean 390.22 µm). One patient did not complete the follow-up and was excluded from the study. The pre- and postoperative best corrected visual acuity are given in Table 1. Data show a slight visual acuity improvement, not a statistically significant (Table 2).

**Table 1:** Best correct visual acuity (BCVA) (LogMAR) preoperatively and at 12-months follow-up

Patient	BCVA	
	Pre-op	12 Months
1	0.0	0.0
2	0.3	0.2
3	0.4	0.4
4	0.1	0.1
5	0.0	0.0
6	0.0	0.0
7	0.3	0.3
8	0.4	0.4
9	0.0	0.0

**Table 2:** Visual acuity (BCVA) (LogMAR), keratometry, corneal thickness and endothelial cells count (mean + standard deviation) at different time points

	Pre-op K1	1 Months K1	3 Months K1	6 Months K1	12 Months K1	*P*
Visual acuity	0.17 ± 0.18				0.15 ± 0.17	NS
K1	46.91 ± 1.9	46.97 ± 1.91	47.07 ± 2.54	47.11 ± 2.21	47.36 ± 2.66	NS
K2	50.75 ± 2.93	51.09 ± 2.71	50.77 ± 3.01	50.63 ± 2.98	50.53 ± 3.35	NS
Corneal thickness	390.22 ± 15.8	365 ± 14.49	355.88 ± 22.52	351.11 ± 17.28	360 ± 25.83	< 0.002
ECD	2362.89 ± 26.14				2394.56 ± 9.51	NS

*ECD* endothelial cells count

**Fig. 1:**
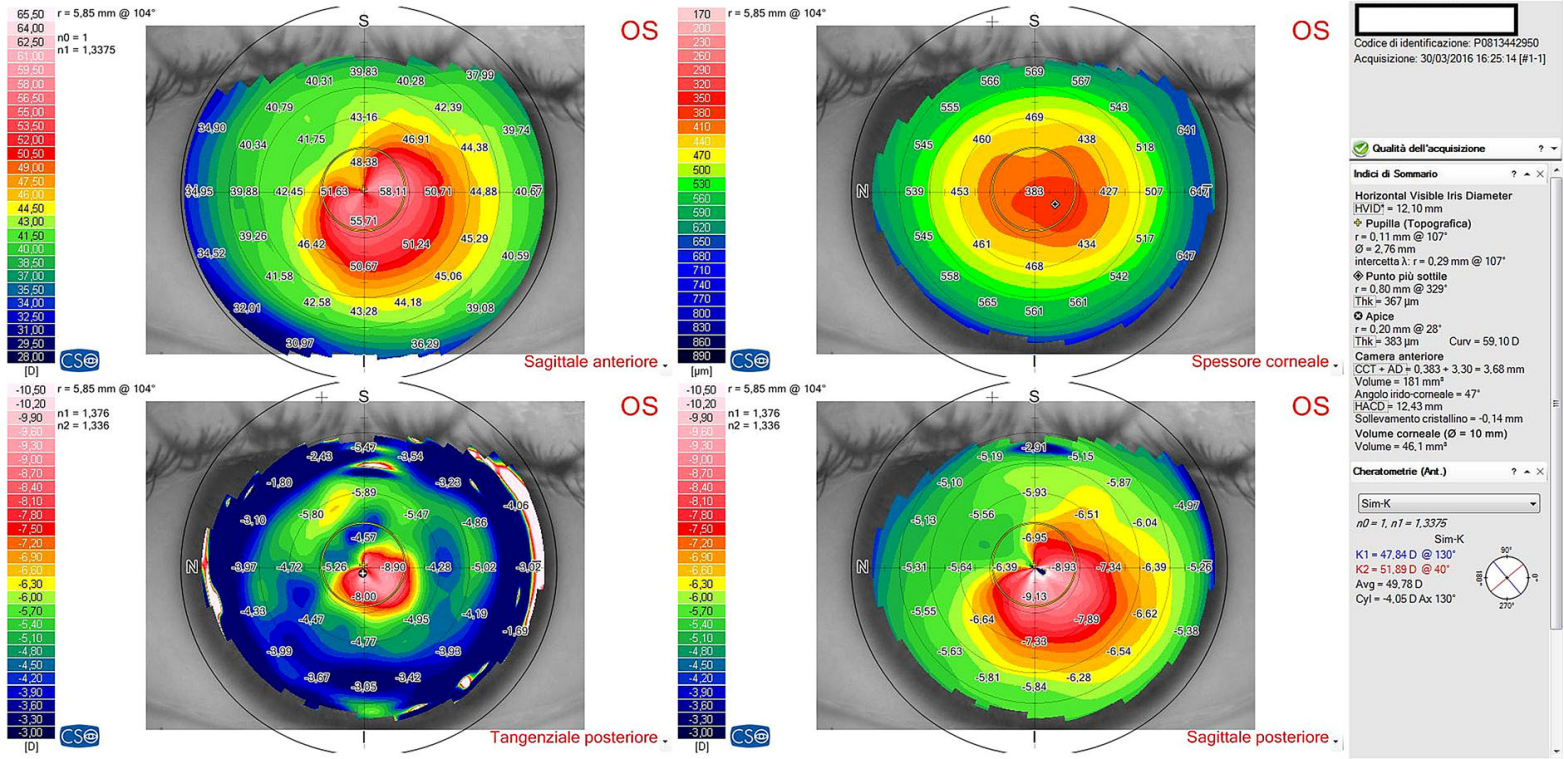
Corneal topography images of a patient preoperatively

**Fig. 2:**
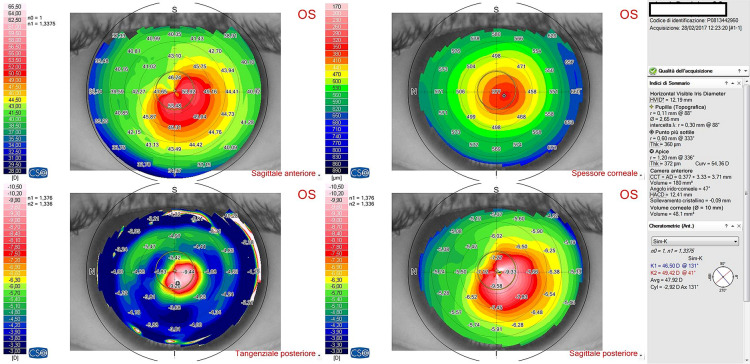
Corneal topography images of a patient at 12 months follow-up after epi-off-stromal lenticule-on corneal collagen cross-linking in thin keratoconic corneas

**Fig. 3:**
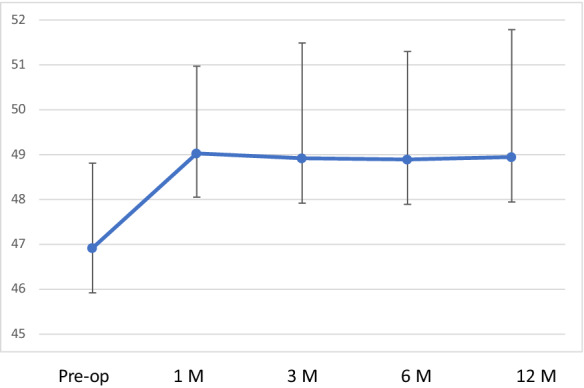
Average keratometry at different time points

**Fig. 4:**
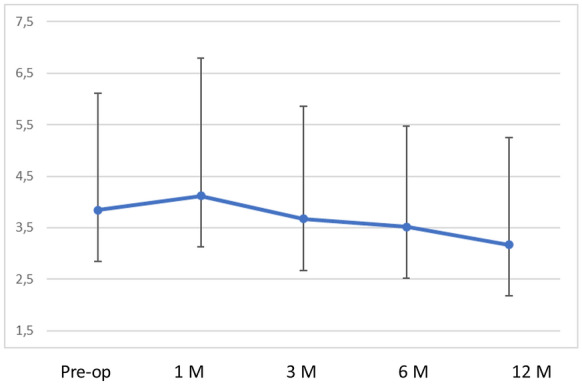
Corneal astigmatism (K2-K1) at different time points

Preoperative mean K1 was 46.91 ± 1.9 dioptres and mean K2 was 50.75 ± 2.93 dioptres, respectively. At 12 months follow-up, mean K1 was 47.36 ± 2.66 dioptres and mean K2 was 50.53 ± 3.35 dioptres, respectively, with no statistically significant difference (*p* > 0.95),); however, at one month follow-up the K values showed some flattening (Table 2, 3 and 4) (Figs. 1, 2, 3 and 4).

**Table 3:** Average keratometry at different time points

	Pre-op	1 Months	3 Months	6 Months	12 Months
1	49.295	49.895	48.865	49.505	49.38
2	51.68	53.025	54.68	54.255	54.985
3	49.865	49.235	48.595	48.1	47.96
4	47.35	47.515	47.37	46.905	45.985
5	46.655	46.945	46.69	46.87	47.4
6	47.11	47.76	47.035	47.23	47.06
7	46.72	47.165	47.055	47.21	47.09
8	50	49.895	51.15	50.905	51.935
9	49	49.89	48.85	48.91	48.74
Mean	46.91	49.04	48.92	48.88	48.95
SD	1.9	1.94	2.57	2.43	2.84

*SD* standard deviation

**Table 4:** Corneal astigmatism (K2-K1) at different time points

	Pre-op	1 Months	3 Months	6 Month	12 Months
1	6.37	7.95	6.61	5.99	6
2	3.64	2.33	1.89	3.05	2.31
3	4.05	3.85	3.33	2.76	2.92
4	1.38	2.87	2.88	2.65	0.69
5	0.93	2.75	2.82	1.44	1.26
6	2.44	1.66	1.61	2.96	3
7	2.64	1.15	1.35	0.96	1.14
8	7.56	7.95	73	6.09	5.31
9	5.56	6.56	5.24	5.78	5.88
Mean	3.84	4.12	3.67	3.52	3.17
SD	2.27	2.67	2.19	1.95	2.08

*SD* standard deviation

**Fig. 5:**
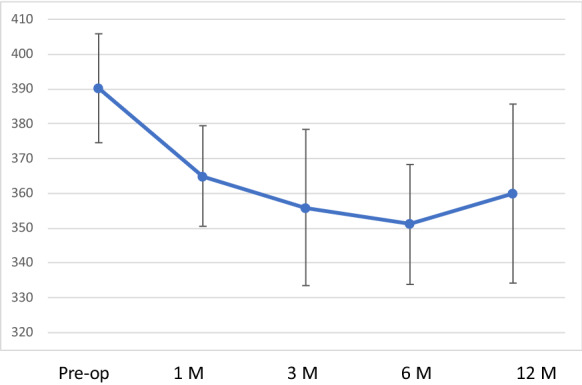
Corneal thickness at different time points

**Fig. 6:**
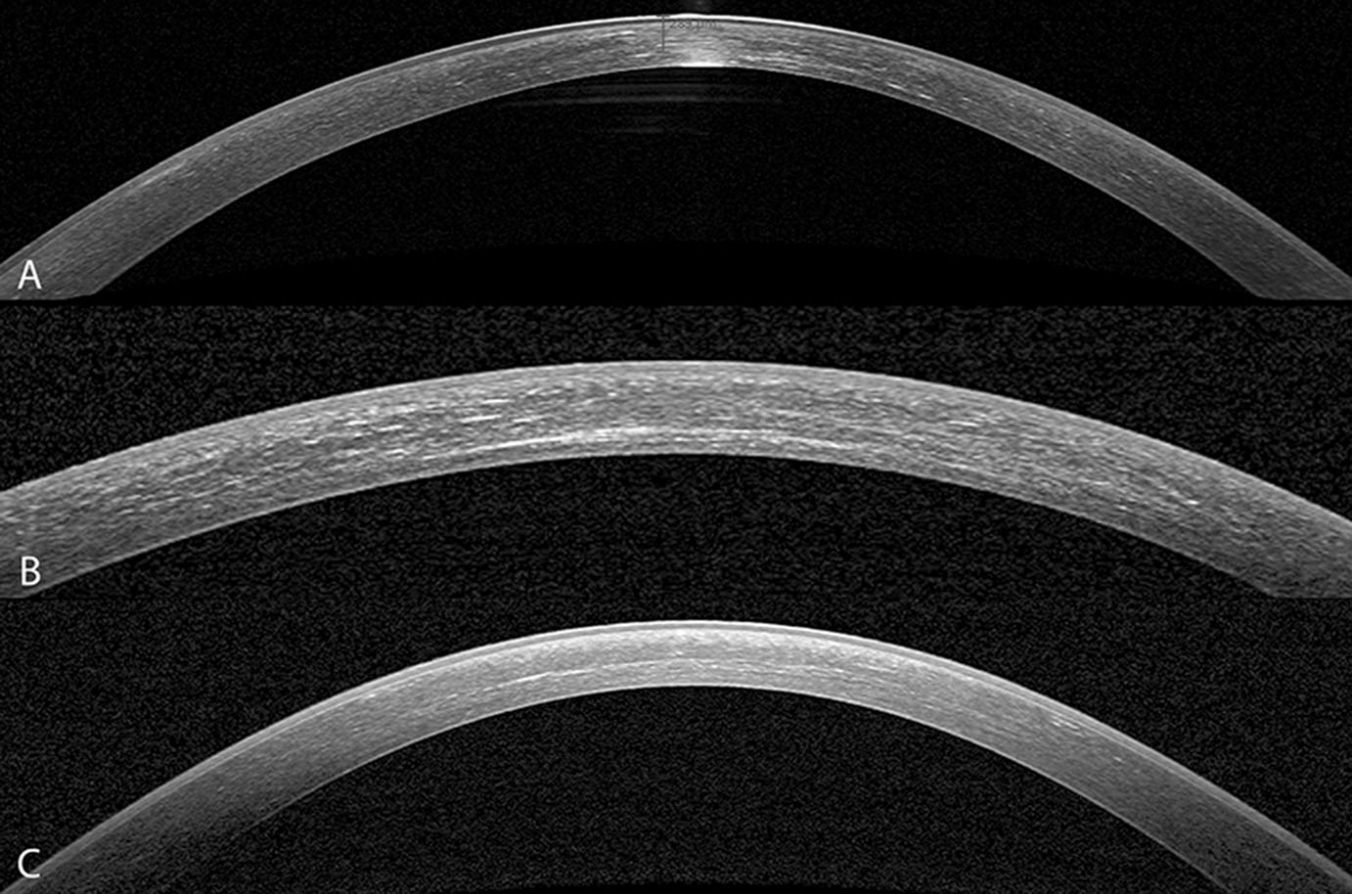
Heidelberg Spectralis optical coherence tomography (OCT) images of 3 different patients clearly showing the demarcation line at 1 (**a**), 3 (**b**) and 6 (**c**) month follow-up after epi-off- stromal lenticule-on corneal collagen cross-linking in thin keratoconic corneas

The mean preoperative thickness at thinnest point was 390.22 µm ± 15.8 µm, and at 12 months, the mean corneal thickness was 360 µm ± 25.83 µm. This was statistically significant with the cornea being thinner than the preoperative value (*p* < 0.002) (Table 2) (Fig. 5). The endothelial cell density remained stable in all patients throughout the follow-up period. The average preoperative value was 2362.89 ± 126.14, and the postoperative value at 12 months was 2394.56 ± 79.51 cells per square millimetre (Table 2). The AS-OCT showed a demarcation line in all cases at 1 and 3 months follow-up (mean depth was 283 µm and 267 µm, respectively), and in 3 cases, it persisted up to 6 months (Fig. 6). IVCM images were acquired from the central cornea and showed a slight subepithelial haze in the healed epithelium at 1-month follow-up, but largely disappeared by 3 months and completely by 6 months. Reduced keratocyte density and stromal edema were observed in the anterior and intermediate stroma immediately after treatment and at 1 month. Keratocyte repopulation was noted in the central treated area, where the edema had disappeared with no difference at 6 and 12 months compared to preoperative.

None of the patients reported any surgery or treatment-related adverse events, the treatment was well tolerated in all patients, and all of them have worn the contact lenses again without any intolerance.

## Discussion

Our study showed the safety and the efficacy of performing an epi-off CXL by overlaying a stromal lenticule of standard thickness (100 µm) and diameter (8.5 mm), obtained by femtosecond laser dissection from a donor cornea after removal of the epithelium. Corneal cross-linking with riboflavin/ultraviolet-A was shown to be a safe and efficient procedure, minimally invasive, and currently the gold standard treatment for progressive KC and other cornel ectasia. Its aim is to increase the biomechanical stability of the stromal tissue and halt the disease progression with the formation of new chemical bonds in the stroma between the extracellular matrix proteins and collagen fibrils [9–11]. The CXL is performed irradiating the corneal stroma with UVA absorbed by the riboflavin [12]. This absorption behaves as a protection against the ultraviolets toward the deeper structures such as the corneal endothelium, lens and retina [13]. The cell damage threshold of UVA-irradiation combined with riboflavin is 10 times higher than with UVA-irradiation alone [14]. In conventional CXL procedure with 0.1% riboflavin in dextran, 20.0% solution and 3 mW/cm^2^ of UVA for 30 min, the treatment parameters are assumed to treat the anterior 300 μm of the corneal stroma, while in thin corneas the risk of damaging deeper structures is high [13–15]. In fact, performing a CXL in corneas thinner than 400 μm the cytotoxicity threshold of 0.35 mW/cm^2^ for the endothelial cell damage can be reached. Kymionis et al. reported a significant postoperative reduction in endothelial cell density in patients with thin corneas (range 340–399 μm) who underwent conventional CXL procedure [16]. Hence, corneal thickness is an important factor to the overall safety of the procedure and only the patients with a de-epithelialized corneal thickness of at least 400 μm can be treated with conventional CXL in all safety [6].

Undoubtedly, the threshold of 400 µm is an important limitation for the standard CXL. In fact, in those patients who wear contact lenses with a good vision with a documented progression of corneal ectasia and a thin cornea (less than 400 µm) the standard CXL cannot be done safely due to the high risk of complications for the deeper structures. These are patients who might progress further over time and a corneal transplant could be the only solution to restore their vision. This is especially true in populations of Asian and African origin with inherently thinner corneas [17, 18].

Several strategies have been employed to over this risk in thin corneas. In transepithelial CXL, the treatment is carried out without epithelial debridement using di enhancers to help riboflavin penetrate to the corneal stroma through the intact epithelium: this strategy adds about 50 microns to the corneal thickness. Moreover, the epithelium acts as a further protection to the endothelium from the toxicity induced by the UVA. Even though some authors have shown the efficacy of transepithelial CXL to halt the progression of keratoconus this technique is not considered as effective as conventional epithelium-off CXL [19–22]. The criticism of this technique is in the variable passage of riboflavin through the intact epithelium and potential uneven absorption of the UVA radiation in the stroma. In corneas that have undergone transepithelial CXL, the postoperative demarcation line depth was only approximately 100 μm, in contrast to about 300 μm in conventional CXL with epithelial debridement and recent studies have shown that the effect of CXL with epi-on treatment is less than with epi-off [9, 19]. Moreover, Wollensak et al. estimated a 64% increase in corneal rigidity in human corneas with transepithelial CXL using topical anesthetics and benzalkonium chloride as enhancers, versus a 320% increase when using CXL with de-epithelialization [23].

A modified CXL technique employed in thin corneas is the custom epithelial debridement technique that was first described by Kymionis et al. in 2009: the authors performed CXL in two ectasic eye with thinnest stroma of less than 400 μm leaving a small, localized area of corneal epithelium at the thinnest area over the apex of the cone [24]. The aim of the authors was to have a shield made by an intact area of epithelium soaked with riboflavin to have UVA attenuation over the thinnest corneal point, while the disepithelized paracentral cornea allowed riboflavin penetration. The results obtained indicate a certain amount of efficacy on stabilising the ectasia, but some authors reported a significant endothelial cell loss after this procedure [25–27].

Another strategy to perform CXL in thin corneas consists in increasing cornea thickness by the use of hypo-osmolar riboflavin or saline prior to UVA exposure. The deepithelialized cornea can swell to double its normal thickness when irrigated with a hypo-osmolar solution [28]. Hafezi et al., as first in 2009, proposed this technique for corneas thinner than 400 microns, and they observed a stabilization of corneal ectasia in 20 eyes treated with this technique, and similar results were later confirmed by other authors [29–31]. However, after this technique was reported permanent corneal scars and permanent stromal scar, and Gu et al. showed a decreased endothelial cell density 3 months after the treatment [32, 33]. Some authors believe that this technique is not reliable and that is important monitoring the corneal thickness throughout CXL treatment with hypo-osmolar riboflavin because artificial cornea swelling effect is transient. Kaya et al. and Soeters et al. measured intraoperative corneal thickness and they found that the thinnest pachymetric values decreased significantly after 10 and 30 min of riboflavin application, with or without UVA irradiation [34, 35]. This was afterwards confirmed by other authors with different concentrations of riboflavin [36, 37]. Moreover, CXL can be expected to have less effect on biomechanics of artificially swollen corneas due to the lower relative concentration of collagen in the hydrated stroma [38]. Thereby, although with this technique in some cases the results were acceptable, clinical data are limited, its safety is uncertain, and it is still not clear whether the swollen corneas obtained with hypo-osmolar riboflavin behave similarly to the non-swollen ectasic corneas [10].

Jacob et al. proposed the use of contact lenses to increase the corneal thickness to perform CXL in thin corneas [39]. They have described results in 14 eyes with 6 months follow-up in which used a daily disposable soft contact lens (14 mm diameter, 8.6 mm basal curvature; 90 µm thickness, made of hilafilcon, no UV filter, Bausch & Lomb) immersed in iso-osmolar riboflavin 0.1% with dextran for 30 min which was placed onto a deepithelialized and riboflavin-saturated cornea. A standard protocol CXL was then performed, and no significant endothelium loss or signs of postoperative endothelial damage were observed. This technique has some advantages which are due to the non-swollen cornea, but also some disadvantages as the surface irradiance at the level of the corneal stroma is reduced by 40–50% due to the absorption by the riboflavin film and by the soaked contact lens. Furthermore, oxygen diffusion might be hindered by the contact lens and the effect of CXL may be reduced. Therefore, more extensive studies are necessary to validate this technique.

Sachdev et al. in 2015 described a technique of stromal expansion in ultra-thin cornea using a donor lenticule obtained after small-incision lenticule extraction (SMILE) for myopic correction [7]. SMILE is a relatively new refractive surgery technique that consists of removing a stromal lenticule, through a single small incision without the flap creation. The authors described 3 patients affected by progressive corneal ectasia and at 6-month follow-up noted a topographic corneal stability, no endothelial damage and a postoperative demarcation line at a depth ranging from 280 to 300 mm. This technique is undoubtedly interesting, but the lenticules were not tailored for the CXL. Lenticules obtained by SMILE surgery have a different thickness in the different points due to its meniscus shape and its thickness and diameter vary with the refractive error of the patient. Moreover, currently there is no other clinical study supporting this method.

In our work, we tried to standardise the technique described by Sachdev using in all cases a corneal stromal lenticule of 8.5 mm diameter and 100 µm of uniform thickness. This is important in order to obtain a proper protection from the irradiance all over the cornea. In our experience, this improvement seems have not changed the efficacy of the treatment as we obtained a stability of the functional indices and the topographic values throughout the 12-month follow-up. Moreover, the demarcation line, as seen by optical coherence tomography, was found at a depth of 283 µm (mean depth at one month) + 100 µm from the surface considering the thickness of the lenticule. These data are consistent with the depth of demarcation line seen with keratoconus corneas treated with standard CXL who do not need the additional lenticule: to our believe, this is an indirect sign of the efficacy and rational of the technique proposed; in fact, the demarcation line is considered to be evidence of the occurrence of cross-linking and its depth indicates the depth of treatment.

As already described by Sachdev, even in our experience we found that the technique is easily executable and repeatable as the lenticule is stable over the deepithelialized cornea and the surgical maneuvers are routinely performed. Also, it is important to underline the safety of the technique as no complication was seen, neither intra- nor postoperatively. In fact, none of the patients developed corneal scars and the endothelial cells count was unremarkable over the entire follow-up.

In our opinion, the efficacy and safety of our treatment, the effectiveness and safety of the treatment we have observed the effectiveness and safety of the treatment constitutes an important advantage over other techniques proposed in the presence of thin corneas, in the treatment of which, as previously discussed, poor efficacy or possible onset of complications have been observed such as decreased corneal transparency or endothelial damage. Furthermore, in our experience all of them return to a normal life as the vision and particularly the contact lens tolerance was preserved.

One limitation of this study is the use of the femtosecond laser in terms of costs and availability. Other limitations are the small patient population and the relatively short follow-up as the corneal ectasia progression is unpredictable. These features limit the statistical value of our data and make this study worth considering as a pilot study: our results need to be confirmed by further prospective studies with larger number of patients.

In conclusion, we treated progressive keratoconus in very thin corneas increasing the corneal thickness by adding a stromal lenticule obtained with femtosecond laser from a donor cornea; in our experience, the technique has shown to be safe, simple and effective of conducting standard CXL. The stromal lenticule should behave similarly to the patient’s stroma being treated and absorb enough UVA preventing the rays from reaching the endothelium or passing through to the lens and retina. For the treatment of ultra-thin corneas, compared to other technique proposed in the past, stromal expansion with a femtosecond obtained lenticule seems to have the advantage of high safety and reproducibility. Thus, having the ability of creating precise and reproducible lenticules, a standard CXL can be performed on patients with progressive KC and good contact lens corrected vision, in order to preserve the vision and reduce the possibility of a future lamellar or perforating transplant.
